# Burn Wound Dynamics Measured with Hyperspectral Imaging

**DOI:** 10.3390/ebj6010007

**Published:** 2025-02-13

**Authors:** Thomas Wild, Jörg Marotz, Ahmed Aljowder, Frank Siemers

**Affiliations:** 1 Clinic for Plastic and Handsurgery and Burn Center, BG-Clinics Bergmannstrost, D-06002 Halle (Saale), Germany; thomas.wild@woundconsulting.de (T.W.); frank.siemers@bergmannstrost.de (F.S.); 2 Clinic of Dermatology, King Hamad University Hospital, Shaikh Isa Bin Salman Causeway, Al Sayh 24343, Bahrain; ahmedaljowder@hotmail.com; 3 Regenerative Medicine, Arabian Gulf University, Road 2904 Building 293 Manama, Manama 329, Bahrain

**Keywords:** burn depth assessment, burn wound dynamics, hyperspectral imaging

## Abstract

**Introduction:** Hyperspectral Imaging (HSI) combined with an augmented model-based data processing enables the measurement of the depth-resolved perfusion of burn wounds. With these methods, the fundamental problem of the wound dynamics (wound conversion or progression) in the first 4 days should be parametrically analyzed and evaluated. **Material and Methods:** From a cohort of 59 patients with burn injuries requiring medical intervention, 281 homogenous wound segments were selected and subjected to clinical classification based on the duration of healing. The classification was retrospectively assigned to each segment during the period from day 0 to day 2 post-burn. The perfusion parameters were presented in two parameter spaces describing the upper and deeper perfusion. **Results:** The investigation of value distributions within the parameter spaces pertaining to four distinct categories of damage from superficial dermal to full-thickness burns during the initial four days reveals the inherent variability and distinct patterns associated with wound progression, depending on the severity of damage. The analysis highlights the challenges associated with estimating the burn degrees during this early stage and elucidates the significance of deeper tissue perfusion in the classification process, which cannot be discerned through visual inspections. **Conclusions:** The feasibility of early classification on day 0 or 1 was assessed, and the findings indicate a restricted level of reliability, particularly on day 0, primarily due to the substantial variability observed in wound characteristics and inherent dynamics.

## 1. Introduction

The treatment of burn wounds continues to face the fundamental challenge of early diagnosis, which is crucial for optimizing treatment effectiveness and outcomes [1,2]. Burn injuries can be categorized into four main classes: superficial, superficial dermal, deep dermal, and full thickness. Based on findings from Hyperspectral Imaging (HSI) analysis of burn measurements [3], these classifications are further refined as follows:

- 2a: Superficial (more severe than a standard sunburn).
- 2b1: Superficial dermal.
- 2b2: Deep dermal.
- 3: Full thickness.

The degrees correspond to an increasing depth of damage (“burn depth”) with increasing degradation of the skin structure, including the microcirculatory perfusion network [4].

Damage up to and including mid-dermal (2a–2b1) normally possesses a sufficient healing potential to be treated conservatively with good results. For deep dermal damage (2b2–3) normally a surgical treatment is necessary to remove necrotic tissue and ensure a healing without difficulties and with good results.

However, burn wounds often show a dynamic development over the first 3–4 days known as “burn wound progression” or “wound conversion” first described by Jackson [5,6,7,8,9,10]: on day 0, using standard clinical methods of wound assessment, the degree of damage can be perceived as rather superficial or medium (2b1) and then develops to a deeper damage (2b2). The intrinsic wound processes in this period are very complex and also not yet sufficiently researched [11,12].

The experience shows that especially for 2nd-degree damage (2b1↔2b2), the usual experience-based and subjectively biased visual estimation of the burn depth at the first 3 days is very unreliable and only yields from the third day onwards a reliable classification [13,14]. Therefore, it is often waited until the damage degree is unmasked on the third or fourth day.

Also available measuring methods like Laser-Doppler-Imaging, LDI [13,15], yield reliable classifications only from the third day on. For a more detailed analysis of the burn dynamics about the first 2–3 days, measurement methods providing more extensive information about the wound state are needed [16].

Hyperspectral Imaging (HSI) is a non-invasive measurement technique primarily used to assess the skin’s perfusion state [17]. Advanced model-based processing of remission spectra allows for depth-resolved visualization of wound perfusion across six layers. This capability is essential for characterizing the extent of damage and estimating the wound’s healing potential. The original methodology is outlined in [3] and summarized in the subsequent chapter.

The study aimed to:

1. Analyze and evaluate the perfusion states of burn wounds and describe their dynamic changes over the initial four days post-burn in relation to damage classes.
2. Assess the feasibility of estimating damage classes on day 0 or day 1.
3. Present analytical methods based on HSI and model-based data processing as a foundation for developing a classification system for burn wounds.

## 2. Materials and Methods

### 2.1. Measurement Method

Hyperspectral Imaging (HSI) is based on optical remission spectroscopy in a spectral range from 450 to 1000 nm. In this range, especially, hemoglobin is the main absorber of light. By multiple scattering of the irradiated white light and the specific absorption of the main components (besides hemoglobin, melanin, collagen, water, and fat), spectrally modified backscattered light (remission) is created, which can be split into the spectral parts by a spectroscopic module and subsequently be detected by an image sensor. In our measuring project, the HSI camera TIVITA (Diaspective Vision GmbH, Am Salzhaff, Germany) was used. A description of the camera system and the measurement procedure can be found in [17].

### 2.2. Data Processing Methods

Theoretically, the volume fractions of skin or wound components can be calculated from remission spectra. These spectra incorporate contributions from spatial structures (layered systems) and biochemical components, such as hemoglobin, collagen, melanin, water, and fat, each exhibiting unique spectral characteristics.

**Figure 1:**
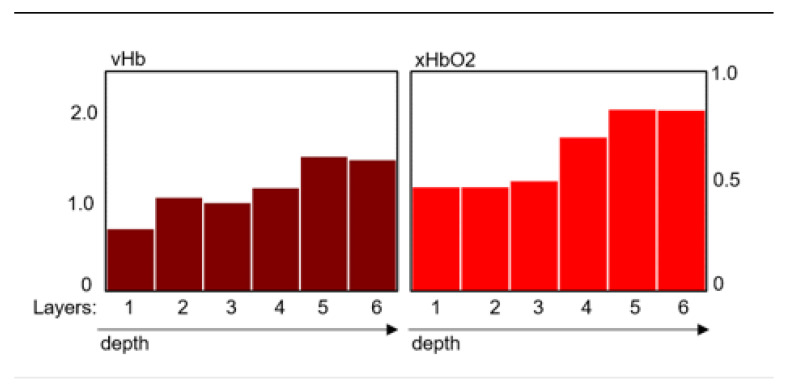
Depicts the so-called perfusion profiles derived from the remission spectra using a six-layer skin model applicable to burn wounds. The profiles represent hemoglobin volume (vHb) on the left and oxygen saturation (xHbO2) on the right. The *x*-axis identifies the skin layers, while the *y*-axis shows vHb as an index (0–1) and xHbO2 as a percentage (0–100). The figure presents an example of a superficial burn segment (2a).

For the specific information retrieval, a 6-layer model of the skin/wound and an approximate solution process for the inverse problem (calculation of the system parameter from the remission spectra) were developed, which allows for the calculation of so-called perfusion profiles [18]. These profiles consist of the two components volume part (vHb) and oxygen saturation (xHbO2) of the hemoglobin for each of the layers. This inverse procedure does not allow for an exact determination of the actual layer depths, so the profiles are accordingly presented as a series of bars with equal width (Figure 1):

For the scaling of the vHb values, depending on the width of the layers, globally fixed scale factors are used. The main benefit of this method is that nearly all of the information of the spectra is transformed uniquely into the model system.

**Figure 2:**
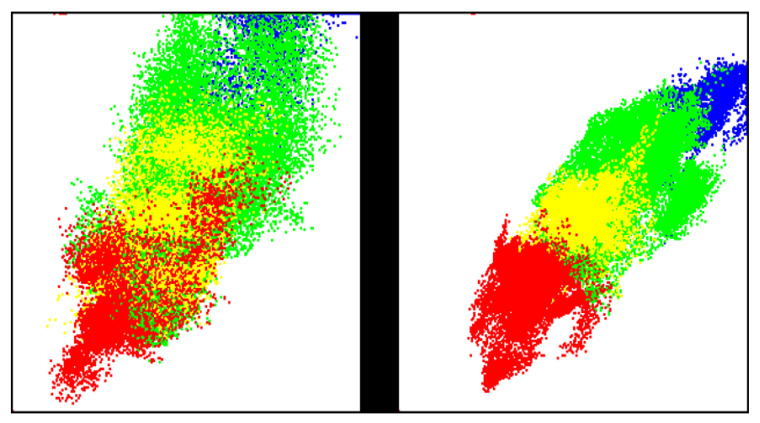
Left: PS_1: vHb_1–xRate, right: PS_2: vHb_2–flow_2, The parameter values range from 0 to 1, left to right and bottom to top; class distributions on day 3 after the burn; classes: 2a: blue, 2b1: green, 2b2: yellow, 3: red; in this 2-dimensional presentation, the higher classes partially mask the lower ones: 3 > 2b2 > 2b1 > 2a. The distributions represent the values for every pixel of all selected segments from the wounds.

From the perfusion profiles, secondary parameters are calculated, describing the composed volume part and the oxygen saturation of hemoglobin in the upper (_1) and deeper (_2) layers (vHb_1,2, xHbO2_1,2). Moreover the blood flow (flow_1 = $\frac{v_{1}\cdot x_{1}}{x_{a}-x_{1}}$, v_1_ = vHb_1, x_1_ = xHbO2_1, x_a_ = arterial oxygen saturation, flow_2 = $\frac{v_{2\cdot \left(x_{2}-x_{1}\right)}}{x_{a}-x_{2}}$, v_2_ = vHb_2, x_2_ = xHbO2_2) through the upper and lower layers, as well as the oxygen consumption rate (xRate = $v_{3}\cdot \left(x_{a}-x_{1}\right)$, v_3_ = volume part in the middle layers 3–4) in the upper layers. In the following, for a clearer presentation of the parameter distributions, two 2-dimensional parameter spaces are used (PS_1,2), exhibiting the best discriminant separation of the burn class distributions (Figure 2):

- PS_1: Superficial perfusion (x: vHb_1, y: xRate)
- PS_2: Deep perfusion (x: vHb_2, y: flow_2)

All parameters have a value range of 0.1.

### 2.3. Clinical Reference Classification

To establish a clinical reference classification, patients with burn wounds treated conservatively were monitored for a two-week period following the initial trauma, during which the extent of wound healing was documented. The time taken for spontaneous wound closure, indicated by the presence of a continuous epithelial cell covering, was used as an objective indicator of burn depth. Segments that achieved spontaneous wound closure by day 14 after injury were categorized as 2a. Category 2b2 was defined as secondary wound closure occurring after 21 days or based on clinical decisions for burn wound excision and grafting. Segments that healed between 14 and 21 days were clinically assigned to category 2b1.

This clinical classification is assigned on day 3 or 4 when a recognizable steady state is reached, as evidenced by distinct patterns observed in the parameter spaces [16], and then retrospectively assigned to the preceding days (day 0 to 3). Wounds clinically classified as being treated surgically are classified as 2b2 in all cases.

All clinical assessments and decisions were carried out by two experienced clinicians, adhering to the clinic’s standard protocols.

The hypothesis to be tested posits that thermal damage initiates a specific burn or damage class that remains consistent but exhibits class-specific wound dynamics over the subsequent 3–4 days. Consequently, these classes are associated with day-specific characteristics that can be described using the parameters.

The following analysis of the wound dynamics primarily concerns classes 2b1 and 2b2 because a superficial 2a-damage and also a deep class 3-damage can be clinically estimated with higher reliability.

### 2.4. Segments as Basic Wound Elements

Hyperspectral Imaging (HSI) provides up to 24 parameters that enable detailed, depth-resolved, and localized characterization of burn wounds [18]. Specialized segmentation techniques, developed through extensive analysis of burn data, divide heterogeneous wound areas into segments that are homogeneous in their parameter values [3,19]. These segments were further tested to ensure each represented a single burn class.

### 2.5. Classification of Wound Segments

Class 2b1 represents positive developments in the initial three days following the burn, while class 2b2 signifies negative developments. This demarcates the shift from conservative treatment (2b1) to surgical intervention (2b2).

Already known results of the analysis of perfusion profiles of burn wounds [3] show characteristic differences in the profile form and changes in time over the first 4 days for the 4 classes. The clinical reference classification exhibits no contradictions concerning discrimination in the classes and in conservative and surgical treatment decisions in our study.

### 2.6. Measurements

A prospective single-center study included patients who met the following criteria: age ≥ 18 years, second- or third-degree burns, written informed consent, and presentation within 24 h of injury [20]. Patients with facial burns, infected wounds, those treated with enzymatic debridement, or those with systemic inflammatory responses were excluded. To minimize systemic influences, only wounds of moderate size were included in the study.

Patients have been treated according to the normal standard at the conducting clinic without surgical intervention before day 3: When the patient is admitted, initially a debridement, removal of blisters, a shave, and wound cleaning with an antiseptic are carried out, followed by a first assessment of the state of wound damage.

The dressing concept is based on national and international guidelines regarding the local treatment of burn patients. As an antiseptic baseline, polyhexanide was used, and exudate management depended on volume (foam till supraabsorber).

Localization, pattern of the injuries, wound assessment, and other relevant data are registered in a documentation system. The wound is dressed with a moist and antiseptic bandage. After 24 h, the re-evaluation of the wound state and assessment is carried out.

The clinical treatment in our clinic was conducted independently of the hyperspectral assessments, and the assessments did not play a role in guiding clinical decision-making. Data were collected on days 0, 1, 2, and 3 following the burn injury. Patients with conservatively treated burn wounds were followed up for two weeks, during which the extent of wound healing was recorded.

A total of 59 patients have been included in the study [20]. The wound segmentation analysis yields 281 homogeneous wound segments with a consistent clinical reference classification.

Statistics

Patients:

Number of patients59Mean age44.15 ± 19.2

Cause of burns:

Scalds32Flames22Explosions5

Localization of the wounds:

Hand19Foot13Lower leg9Thigh7Forearm3Upper arm3Torso front5

Wounds:

Segments per woundMean: 4.6Wound area50–175 cm^2^, mean: 82.4 cm^2^Segment area1.5–20 cm^2^, mean: 3.4 cm^2^

The selected wound segments were assessed to ensure they remained homogeneous upon clinical inspection and were of adequate size to minimize the influence of the surrounding wound areas.

## 3. Results

### 3.1. Wound Dynamics

**Figure 3:**
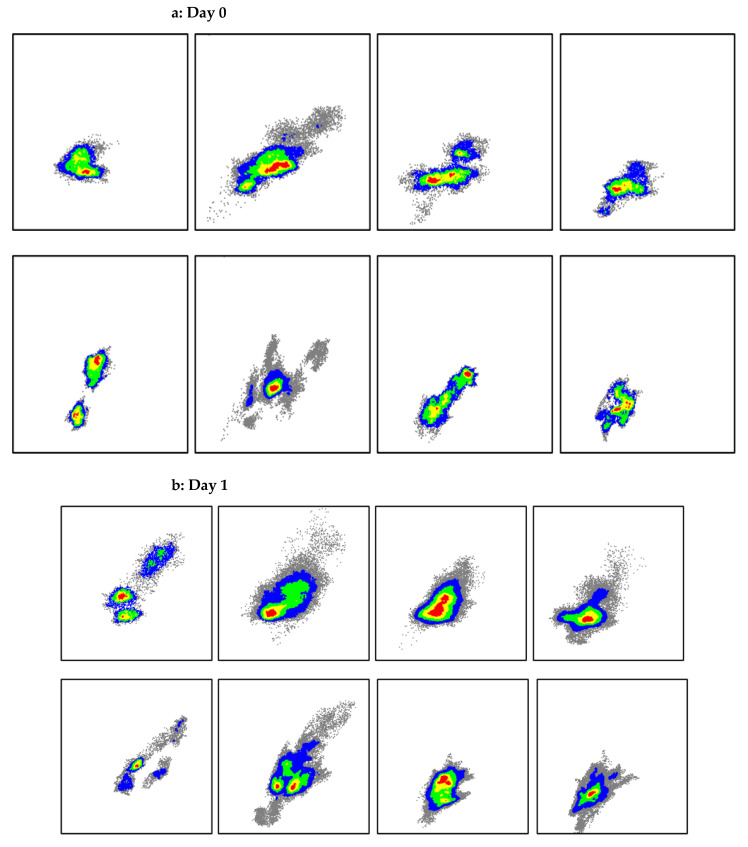

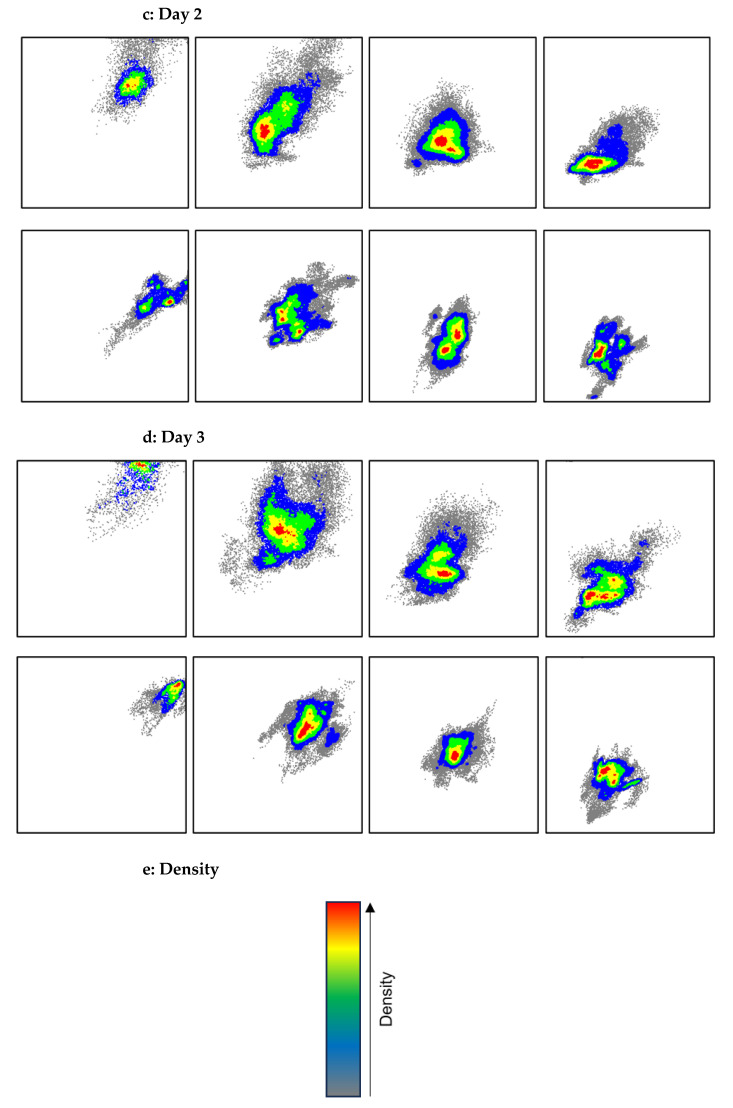
Class distributions in the parameter spaces PS_1 and PS_2 on day 0 (**a**), 1 (**b**), 2 (**c**), and 3 (**d**); first row: PS_1, second row: PS_2; classes from left to right are: 2a, 2b1, 2b2, 3; the colors indicate distribution density ranging from gray to red (**e**).

**Figure 4:**
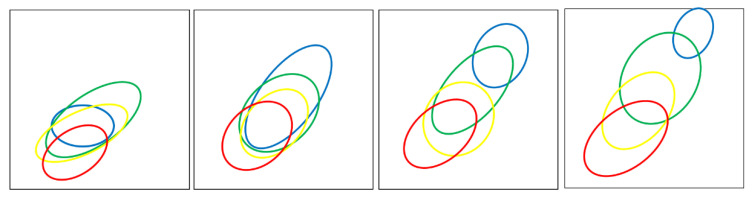
Illustrates abstracted class areas for upper perfusion (PS_1) over four days (day 0 to day 3 from left to right). Class colors are as follows: 2a (blue), 2b1 (green), 2b2 (yellow), and 3 (red). Colored ellipses delineate class boundaries, derived from density distributions shown in Figure 3, using a simplified convex hull approach.

**Figure 5:**
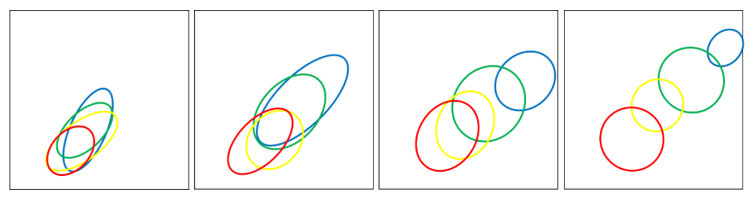
Abstracted class areas for deep perfusion (PS_2) on day 0, 1, 2, and 3 (from left to right); the classes are represented as follows: 2a: blue, 2b1: green, 2b2: yellow; 3: red.

**Figure 6:**
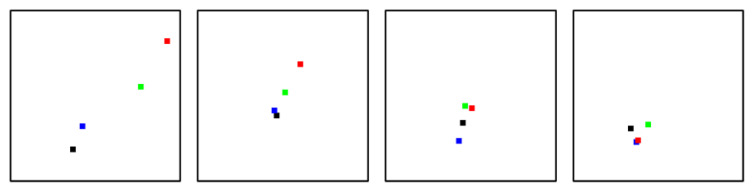
Examples of segment dynamics in PS_2 (deep perfusion) for the classes 2a, 2b1, 2b2, and 3 (from left to right); day 0: black, day 1: blue, day 2: green, day 3: red; measured at wounds at femur and feet.

Each 2D-parameter space depicted in Figure 2, Figure 3, Figure 4, Figure 5 and Figure 6 represents either superficial perfusion (PS_1) or deep perfusion (PS_2). Color-coded distributions illustrate the values of all selected wound segments. Figure 4 and Figure 5 show abstracted distribution areas derived from a convex hull of kernel class regions from the original distributions in Figure 3. By day 3, these distributions become compact and well-separated, particularly in PS_2 (deep perfusion) (Figure 3d and Figure 5).

For a better overview, Figure 4 and Figure 5 schematically show the main distribution areas of the four classes for the upper (Figure 4) and deeper perfusion (Figure 5), depicting the kernel class areas corresponding to Figure 3. The class dynamics in the upper layers (Figure 4) are principally similar to those in the deeper layers (Figure 5), but the distribution areas are larger for all classes, as well as the overlaps of the class areas.

The course of the distributions for single classes in PS_2 (deep perfusion, Figure 5) over several days shows:

- Initially, on day 0, a compact global distribution in the lower left area of the parameter space with strongly overlapping classes; in the following days the distribution subsequently spreads towards the upper right area with reduced overlaps of the classes (increased class discrimination).
- The individual classes show significantly different dynamics over several days:
  - 2a: initially medium values, then a strong movement to the top right and a widening of the class area on day 1; the parameter value variance on day 0 seems to be larger than for the other classes; the final distribution on day 3 is significantly reduced;
  - 2b1: initially some lower values than 2a, a strong movement to the top right with a strong widening on day 1; the main part of the distribution remains underneath the 2a area;
  - 2b2: initially lower values than 2b1; significant lower dynamic than 2b1, no widening of the area; overall, the smallest distribution area;
  - 3: nearly stationary with a widening to the bottom left (=lower values).
- The class dynamics not only lead to a shift in the mean values but also to a widening of the distribution areas (increasing variability).
- Only from day 2 does an increasing discrimination of the classes occur, whereby 2b2 is still widely covered by 2b1 and 2a.
- On day 3 the classes are relatively well separated in PS_2.

Wound physiological interpretation (deep perfusion):

- The hyperemic reaction of the wound is expressed by an increasing vHb_2 and increasing flow_2;
- 2a: The initial reaction is limited; the hyperemic reaction develops over the next 3 days, also significantly strong in the upper layers, because the capillary perfusion is still widely intact;
- 2b1: Initially similar values as 2a, a significant dynamic over several days in the form of a hyperemic reaction, which is also still recognizable in the upper layers, which means the damage is still limited;
- 2b2: Initially on average lower values than 2b1, afterwards a limited hyperemic reaction, clearly lower than with 2b1-wounds; conspicuous the clearly smaller distribution area compared to 2b1, maybe due to the limited dynamic development;
- 3: The perfusion is strongly reduced due to the deep layer damage and decreases further over several days.

On day 0 the classes strongly overlap; a discrimination between 2b1 and 2b2 seems not possible. On day 1, 2a and 2b1 start to separate from 2b2 and 3.

Figure 6 shows examples of the segment dynamics for the 4 classes in PS_2. In this example, the 2a segment starts with relatively low values but shows a strong increase on days 2 and 3. The hyperemic development of the 2b2 is more limited than for 2b1.

**Figure 7:**
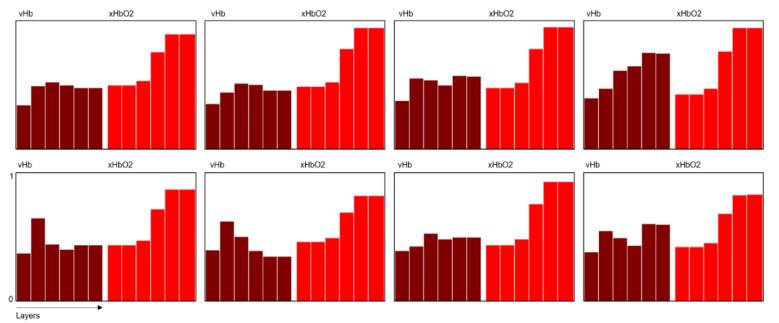
Typical perfusion profiles for class 2b1 (upper row) and 2b2 (lower row) for day 0 to 3 (left to right), measured at wounds at lower legs and hands.

Figure 7 shows typical perfusion profiles for a 2b1 and a 2b2 segment over days 0 to 4. Characteristic of 2b2 (lower row) is the vHb-peak at the upper layer 2 or 3, describing a blood congestion underneath the capillary system due to the damage of the latter. The final xHbO2 values for 2b2 are lower than for 2b1.

### 3.2. Classification

This article outlines a foundational methodology for analyzing burn class dynamics using HSI and spectral data processing. Developing a robust classification system for burn wounds remains a goal for future studies. Particular emphasis is given to distinguishing between 2b1 and 2b2 classes, as this distinction significantly impacts treatment decisions. Comparing Figure 4 (superficial perfusion) and Figure 5 (deep perfusion) underscores the importance of deep perfusion in burn classification.

Qualitative analysis of class discrimination in PS_2 demonstrates that differentiation between 2a and 2b1 and 2b2–3 is somewhat achievable by day 1, albeit with some uncertainty. On day 0, the extensive overlap of distributions hinders reliable classification. Discrimination improves markedly by day 2, and by day 3, the class distributions stabilize with clear separation.

**Figure 8:**
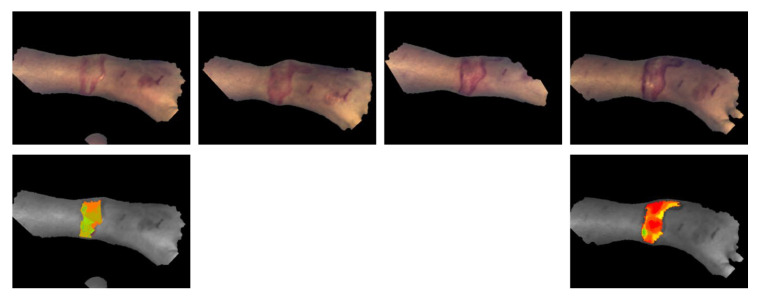
An example of a burn wound on the left foot, caused by scald; from left to right: day 0 to day 3; the preliminary classification by the software is shown for day 0 and day 3 (blue: 2a, green: 2b1, yellow: 2b2, red: 3).

Figure 8 shows an example of a burn wound on the left foot, caused by scald from day 0 to day 3. A preliminary classification has been performed based on the actually available amount of data. The classification on day 3 conforms with clinical reference classification. The classification on day 0 is a little bit less deep, but, as mentioned, with a high uncertainty.

## 4. Discussion

Numerous articles have addressed the early dynamics of burn wounds, examining cellular and molecular processes [5,6,7,8]. However, due to the absence of objective measurement techniques capable of capturing relevant wound parameters during the initial period (day 0 to 3 post-burn), these intrinsic processes cannot be observed to facilitate treatment decisions, such as choosing between conservative or surgical approaches.

Assessments of Laser Doppler Imaging (LDI) accuracy for burn depth evaluation have been conducted at various time points (days 0, 1, 3, and 5). The accuracy of LDI consistently surpassed that of clinical assessment, but its efficacy was limited to 54% on day 0, 79% on day 1, and 95% on day 3 [13]. These findings align qualitatively with our own results, considering the adoption of distinct class definitions.

HSI offers the potential to obtain extensive information about burn wounds, particularly on a macroscopic level, primarily through the assessment of perfusion parameters.

The analysis of the wound dynamics, described by this parameter, exemplifies the fundamental problem of an early estimation of the degree of damage of burn wounds. Figure 5 clearly shows the development of the class distributions over the first four days from a compact global distribution area on day 0 until an untangled distribution with good discrimination of the classes on day 3. This corresponds to the clinical experience that classifications (2b1–conservative and 2b2–surgical treatment) generally are not reliable until day 3.

With a mostly visual inspection of the wound, only superficial features are recognizable (Figure 4), with a limited discrimination of 2b1 and 2b2 also on day 3. The “visual border” tends to run in the upper part of the 2b2 distribution (yellow), so that a larger part of the 2b1 distribution (green) may be wrongly estimated as 2b2. This correlates with our experience that, with the use of a more objective measuring method, which also captured the deeper perfusion (like LDI and HSI), the surgical treatments are reduced in favor of conservative treatments.

Several factors influence parameter distributions:

1. Statistics: The study includes a majority of burns localized to the hands and feet due to the random distribution of burns in the measuring period, limiting generalizability to other body regions. A detailed analysis by wound location was not feasible, and the dataset size was insufficient for statistical validation. However, the study’s primary objective was to demonstrate the data processing methodology.
2. Measurement and Data Processing: The measurement method cannot precisely determine skin or wound depth structure (e.g., layer thickness) [18]. As a result, depth profiles represent relative values based on globally fixed scale factors, leading to variability in parameter distributions.
3. 2D Parameter Spaces: Projections onto 2D parameter spaces (PS_1 and PS_2) simplify class distribution visualization. While higher-dimensional spaces may enhance discrimination, they require significantly larger datasets.
4. Clinical Reference Classification: Healing time-based clinical classifications are inherently imprecise but are supported here by parameter-based classifications on day 3. A universally valid analysis would require standardized burn wound treatment protocols.

The transition towards a positive or negative development, particularly in 2b1-segments, becomes evident from day 0 to day 1, possibly originating from initial states that are indecisive—contrary to the hypothesis—or from initial classes that are indistinguishable based on the HSI parameter on day 0.

The distributions presented in Figure 4 clearly demonstrate that a clinical estimation on day 0 has minimal chances of correctly identifying a deep 2b2-degree, and there is a high probability of misinterpretations as 2b1 at this early stage.

Discriminating between the distributions of different classes, as an indicator of classification quality, is insufficient for a reliable early assessment of burn class on day 0. The classes exhibit significant overlap on day 0; however, Figure 5 illustrates distinct developments, with 2a–2b1 and 2b2–3 becoming significantly separated as early as day 1.

The significant overlap of class distributions on day 0 is primarily due to the global variability of wound characteristics and intrinsic dynamics, which cannot be fully captured by HSI perfusion parameters. Currently, no alternative measurement methods offer more specific wound-related parameters, limiting progress in distinguishing between 2b1 and 2b2 at this stage.

However, class developments on day 1 suggest that early separation of damage classes can be instrumental in determining conservative or surgical treatment options with acceptable reliability.

## 5. Conclusions

HSI provides a practical and detailed understanding of burn wound conditions. It facilitates parametric analysis of wound dynamics based on the degree of damage, offering new insights into burn classification during the initial four days. Key contributions include:

- HSI enables parametric analysis of burn class dynamics using physiologically interpretable perfusion parameters.
- Deep perfusion is crucial for distinguishing burn classes, with HSI being the only method capable of differentiating between superficial and deep injuries.
- The first 24 h are identified as critical for class development, though the processes driving these changes cannot be captured with HSI.

## Data Availability

The original contributions presented in this study are included in the article. Further inquiries can be directed to the corresponding author(s).
